# Artificial Intelligence‐Enabled Electrocardiogram Screening for Detection of Low Ejection Fraction in Routine Care: A Systematic Review and Meta‐Analysis of Randomized Controlled Trials

**DOI:** 10.1111/anec.70248

**Published:** 2026-10-28

**Authors:** Ali Abdelhaleem Omar Ahmed, Omer Abdalhaleem Omer Ahmed, Rana Abdelhaleem Omar Ahmed, Afrah S. Dishean, Collins Gilbert, Mohamed Mirghani Ali Mohamed, Yara Ahmed, Mohamed Hashim Abdelmageed, Ahmed Ashraf Eissa

**Affiliations:** ^1^ Faculty of Medicine National Ribat University Khartoum Sudan; ^2^ Faculty of Medicine University of Khartoum Khartoum Sudan; ^3^ St. George's University St. George's Grenada; ^4^ Faculty of Medicine University of Medical Sciences & Technology (UMST) Khartoum Sudan; ^5^ Cardiac Academy Ain Shams University Cairo Egypt

**Keywords:** artificial intelligence, ejection fraction, electrocardiography, left ventricular dysfunction, meta‐analysis

## Abstract

**Background:**

Left ventricular systolic dysfunction (LVSD) is common and treatable but often undiagnosed, especially in early stages with non‐specific symptoms. AI applied to standard ECGs offers low‐cost screening for low ejection fraction (EF), yet its real‐world impact on diagnosis remains unclear.

**Methods:**

A systematic review and meta‐analysis of RCTs assessed AI‐ECG screening for low EF in clinical practice. The protocol was registered with PROSPERO (CRD420261287652). Databases (PubMed, Scopus, Cochrane Central, Google Scholar) were searched from inception to January 18, 2026, for RCTs comparing AI‐ECG screening to usual care in adults without known heart failure undergoing routine ECGs. The primary outcome was new low EF (≤ 50%) confirmed by echocardiography. Secondary outcomes included echocardiography referral rates, positive predictive value, and cardiology referral rates. Pooled risk ratios (RR) with 95% CIs were calculated using a random‐effects model.

**Results:**

Three RCTs were included (two individual and one cluster) with 37,467 participants. After adjusting for clustering, AI‐ECG screening significantly improved detection of new low EF diagnoses compared to usual care (RR 1.36, 95% CI 1.12 to 1.64; *p* = 0.002; *I* ^2^ = 0%), with a number needed to screen of 204. No significant difference was found in echocardiography referral rates (RR 1.02, 95% CI 0.96 to 1.09; *p* = 0.55; *I* ^2^ = 36%). The PPV of the AI tool ranged from approximately 20% to 34% across studies.

**Conclusion:**

AI‐ECG screening added to routine care improves low EF detection without substantially increasing echocardiography referrals. These findings support AI‐ECG for earlier LVSD detection in at‐risk populations, though further validation across diverse AI platforms is warranted.

## Graphical abstract

Asymptomatic left ventricular systolic dysfunction is common but underdiagnosed. This meta‐analysis of 3 randomized controlled trials (37,467 participants) evaluated AI‐ECG screening for low ejection fraction (EF ≤ 50%). AI‐ECG significantly increased detection of new low EF (RR 1.35, 95% CI 1.15–1.57; *p* = 0.0002) without significantly affecting echocardiography referrals (RR 1.03, 95% CI 0.96–1.09; *p* = 0.45). These findings support AI‐ECG as a scalable tool for earlier detection of a treatable precursor to heart failure.

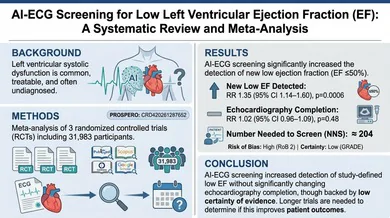

**Abbreviations**
AI: Artificial Intelligence
CI: Confidence Interval
ECG: Electrocardiogram
EF: Ejection Fraction
EHR: Electronic Health Record
FDA: Food and Drug Administration (U.S.)
GRADE: Grading of Recommendations, Assessment, Development, and Evaluations
HFrEF: Heart Failure with Reduced Ejection Fraction
LV: Left Ventricular/Left Ventricle
LVEF: Left Ventricular Ejection Fraction
LVSD: Left Ventricular Systolic Dysfunction
PPV: Positive Predictive Value
PRISMA: Preferred Reporting Items for Systematic Reviews and Meta‐Analyses
PROSPERO: International Prospective Register of Systematic Reviews
RCT: Randomized Controlled Trial
RoB: Risk of Bias
RR: Risk Ratio
TTE: Transthoracic Echocardiogram

## 1 Introduction

Heart failure with reduced ejection fraction (HFrEF) is a major global health issue. It leads to significant illness, death, and healthcare costs (Savarese and Lund 2017; Groenewegen et al. 2020). A major challenge in addressing this problem is the high number of people with undiagnosed left ventricular systolic dysfunction (LVSD). This condition often remains unrecognized until symptoms develop or a serious event occurs (Ammar et al. 2007; Wang, Evans, et al. 2003), yet evidence suggests that early treatment in the asymptomatic or minimally symptomatic phase can improve outcomes (Heidenreich et al. 2022; McDonagh et al. 2021).

Echocardiography is the best method for measuring ejection fraction, but it is not practical for widespread screening due to costs, limited resources, and the need for specific expertise (Ponikowski et al. 2016). The common 12‐lead electrocardiogram (ECG) has been a routine tool in clinical care for many years, but it has had limited success in detecting conditions like LVSD (Wang, Asinger, and Marriott 2003). The rise of artificial intelligence (AI), especially deep learning, has opened new possibilities. AI can analyze subtle patterns in standard ECG readings that are hard for people to notice. These AI algorithms can serve as powerful digital markers for heart problems (Attia et al. 2019; Siontis et al. 2021). Early studies showed that AI‐ECG models could accurately identify patients with low ejection fraction in retrospective datasets (Yao et al. 2021; Cohen‐Shelly et al. 2021).

However, a crucial question remains unanswered: Does using an AI tool in clinical settings improve patient outcomes? Does providing clinicians with this predictive information lead to more diagnoses, more appropriate follow‐up tests, and better overall care? While diagnostic accuracy is important, it is not enough; we need to show that it works in the messy reality of everyday medical practice (Topol 2019). To address this key question, we conducted a systematic review and meta‐analysis of randomized controlled trials. Our aim was to determine whether screening with an AI algorithm for low ejection fraction in adults undergoing routine ECGs results in more new diagnoses of LVSD than standard care. To our knowledge, this is the first meta‐analysis to specifically synthesize evidence from randomized trials on this intervention. While each of the three published RCTs has individually demonstrated positive results, a meta‐analysis offers additional value by providing a more precise pooled effect estimate, examining consistency across diverse settings, evaluating secondary outcomes with greater power, and quantifying heterogeneity to assess the robustness of findings.

## 2 Methods

### 2.1 Search Strategy

A systematic search of the literature was conducted in PubMed, Scopus, Cochrane Central, and Google Scholar from inception to January 18, 2026, in accordance with PRISMA 2020 guidelines. The protocol for this systematic review was registered with PROSPERO (PROSPERO Registration Number, CRD420261287652).

The search strategy was developed using the PICO (Population, Intervention, Comparison, Outcome) framework. It included the following combinations of keywords and Medical Subject Headings (MeSH):(“Artificial Intelligence”[Mesh] OR “Machine Learning”[Mesh]) AND (“Electrocardiography”[Mesh] OR ECG[tiab]) AND (“Stroke Volume”[Mesh] OR “Ventricular Dysfunction, Left”[Mesh] OR “ejection fraction”[tiab]) AND (“Randomized Controlled Trial”[pt] OR randomized[tiab]). The search was limited to studies involving humans. The reference lists of included articles and relevant reviews were screened to identify additional eligible studies. The study selection process is outlined in the PRISMA 2020 flow diagram (Figure 1).

**FIGURE 1:**
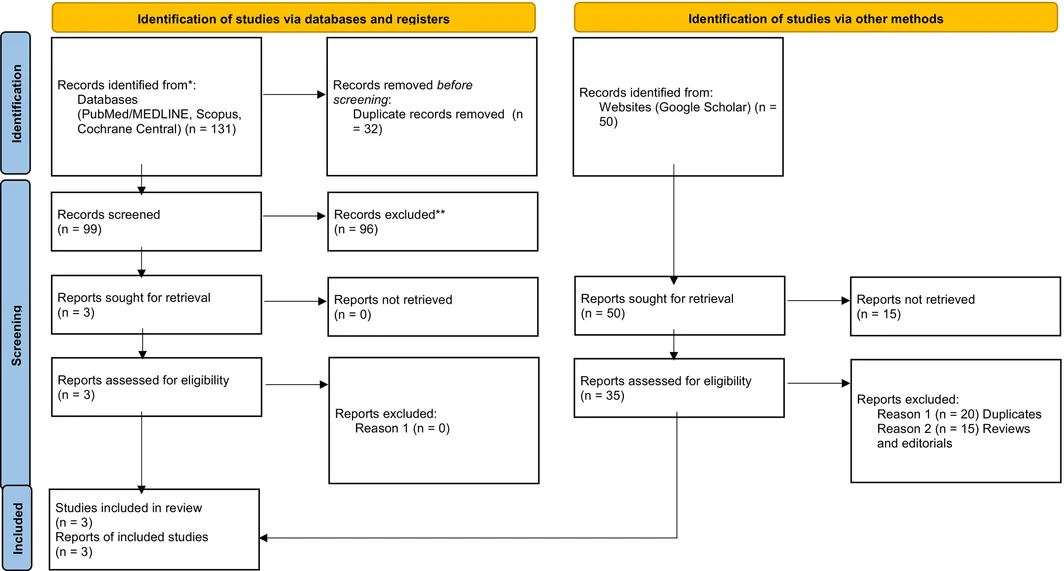
PRISMA flow diagram. Duplicates were removed in two stages: 32 duplicates from 131 database records, and 20 duplicates from 50 gray literature/registry records. * The search was updated on September 20, 2026, using the same strategy. No new eligible RCTs were identified. Two ongoing RCTs (AIM ECG‐AI, NCT05867407; ELEGANT) have not yet reported results. *Consider, if feasible to do so, reporting the number of records identified from each database or register searched (rather than the total number across all databases/registers). **If automation tools were used, indicate how many records were excluded by a human and how many were excluded by automation tools. *Source:* Page MJ, et al. BMJ 2021;372:N71. doi:10.1136/bmj.n71, The PRISMA 2020 statement: An updated guideline for reporting systematic reviews. This work is licensed under CC BY 4.0. To view a copy of this license, visit https://creativecommons.org/licenses/by/4.0/.

### 2.2 Eligibility Criteria

Eligible studies were randomized controlled trials (RCTs), including cluster‐RCTs, involving adult patients (≥ 18 years) without a prior known diagnosis of heart failure or left ventricular systolic dysfunction, who underwent a standard 12‐lead electrocardiogram as part of routine clinical care. The intervention was screening with an AI algorithm applied to the ECG to identify a high probability of low ejection fraction (EF ≤ 50%), with the result made available to the treating clinician. The comparator was usual care (standard ECG interpretation without AI‐based decision support).

The primary outcome was the proportion of patients with a new diagnosis of low EF (≤ 50%) confirmed by echocardiography within 90 days of the index ECG. Secondary outcomes included: (1) the proportion of patients referred for echocardiography, (2) the positive predictive value (PPV) of the AI tool among those who received echocardiography, and (3) the proportion of patients referred to cardiology or a heart failure specialist.

Studies were excluded if they were not RCTs, involved a pediatric population, used AI for purposes other than low EF screening from a standard ECG (e.g., arrhythmia detection), or did not report any of the pre‐specified outcomes.

### 2.3 Data Extraction and Management

Two independent reviewers screened titles, abstracts, and full‐text articles for eligibility. Data from each included trial were extracted using a standardized data extraction form. A third reviewer was available to resolve any discrepancies through discussion and consensus.

The extracted data included:

- Study characteristics (first author, year, design, country, setting)
- Participant characteristics (sample size, age, sex)
- Intervention details (AI algorithm, workflow for delivering results, follow‐up period)
- Outcomes data (event counts and totals for intervention and control groups for all pre‐specified outcomes)
- The characteristics of the included trials are summarized in Table 1. All extracted data were verified against the original publications for accuracy.

**TABLE 1:** Characteristics of included randomized controlled trials.

Study (Author, Year)	Country & setting	Design	Sample size (Intervention/control)	Population	AI‐ECG intervention	Primary outcome definition	Follow‐up for primary outcome
Yao et al. (2021)	USA; primary care & hospital clinics	Cluster RCT (120 care teams)	22,641 (11,573/11,068)	Adults ≥ 18 years without known HF undergoing routine ECG	AI‐ECG result integrated into the EHR as an alert to the clinician	New diagnosis of low EF (≤ 50%) within 90 days of index ECG	90 days
Tsai et al. (2025)	Taiwan; hospital inpatients	Individual RCT	13,631 (6840/6791)	Hospitalized adults without known HF or prior low EF	AI high‐risk flag triggered a real‐time EHR alert for non‐cardiology clinicians	New diagnosis of low EF (≤ 50%) within 30 days of index ECG	30 days
Adedinsewo et al. (2024)	Nigeria; antenatal/postnatal clinics (6 hospitals)	Individual RCT	1232 randomized; 1195 analyzed (587/608)	Pregnant/postpartum women without known cardiomyopathy	Digital stethoscope +12‐lead AI‐ECG; 12‐lead AI results provided asynchronously	Diagnosis of LVSD (LVEF < 50%) during the perinatal period	Through 15 May 2024 (18‐month study period)

Abbreviations: AI, artificial intelligence; ECG, electrocardiogram; EF, ejection fraction; EHR, electronic health record; HF, heart failure; LVEF, left ventricular ejection fraction; LVSD, left ventricular systolic dysfunction; RCT, randomized controlled trial.

### 2.4 Quality Assessment

The methodological quality of each included randomized controlled trial was independently assessed by two reviewers using the Cochrane Revised Risk of Bias tool for randomized trials (RoB 2). This tool evaluates bias across five domains: (1) the randomization process, (2) deviations from the intended interventions, (3) missing outcome data, (4) measurement of the outcome, and (5) selection of the reported result. Disagreements between reviewers were resolved through discussion or consultation with a third reviewer. An overall judgment of “low risk,” “some concerns,” or “high risk” of bias was assigned to each study based on these domain‐level assessments.

### 2.5 Statistical Analysis

All statistical analyses were performed using Review Manager (RevMan) version 5.4. For dichotomous outcomes (new low EF diagnosis, echocardiography referral), pooled risk ratios (RR) with 95% confidence intervals (CI) were calculated using the Mantel–Haenszel method with a random‐effects model.

One included trial [11] was a cluster‐randomized design. To account for clustering, we obtained the intracluster correlation coefficient (ICC = 0.0017) reported in the original publication. With 45 clusters and an average cluster size of 503 patients, we calculated the design effect as 1 + (average cluster size—1) × ICC = 1.8534. The effective sample sizes and event counts for this trial were adjusted by dividing the original numbers by the design effect, as recommended by the Cochrane Handbook. These adjusted values were used in the primary meta‐analysis.

Heterogeneity across trials was assessed using Cochran's Q (*χ* ^2^) test and quantified by the *I* ^2^ statistic. *I* ^2^ values of 25%, 50%, and 75% were interpreted as indicating low, moderate, and substantial heterogeneity, respectively. The statistical significance level was set at *p* < 0.05.

A pre‐specified sensitivity analysis for the echocardiography referral outcome was performed by excluding the trial [20] in which all intervention‐arm participants received a protocol‐mandated baseline echocardiogram, as this did not represent a clinical referral decision in routine care.

### 2.6 Certainty of Evidence Assessment

The certainty of evidence for each prespecified outcome was evaluated using the Grading of Recommendations, Assessment, Development, and Evaluations (GRADE) framework. This structured approach assesses evidence across five domains: risk of bias, inconsistency, indirectness, imprecision, and publication bias. Two reviewers independently performed the GRADE assessments, and any discrepancies were resolved through discussion to reach consensus. Judgments for each domain were synthesized to produce an overall certainty rating (high, moderate, low, or very low) for the effect estimates derived from the meta‐analyses.

## 3 Results

### 3.1 Study Selection

A systematic search of electronic databases (PubMed, Scopus, and the Cochrane Central Register of Controlled Trials) identified 131 records. After removing 32 duplicates, we screened 99 records based on title and abstract. We excluded 96 of these, and assessed 3 full‐text articles for eligibility.

A separate Google Scholar search returned 50 records. After removing 20 duplicates, we screened 30 records. We excluded 15 of these as reviews or editorials. The remaining 15 articles could not be accessed in full text.

So, we assessed 3 full‐text articles from the database searches for eligibility, and all 3 met the inclusion criteria. No studies were excluded at the full‐text stage. The study selection process is summarized in the PRISMA 2020 flow diagram (Figure 1).

### 3.2 Study Characteristics

A total of three randomized controlled trials (RCTs) were included, involving 37,467 participants (Tsai et al. 2025: 13,631; Yao et al. 2021: 22,641; Adedinsewo et al. 2024: 1195). Two trials were individual‐patient RCTs [14,20], and one was a cluster RCT [11]. The trials took place in different settings: primary and hospital care in the United States (Yao et al. 2021), hospital care in Taiwan (Tsai et al. 2025), and antenatal/postnatal care in Nigeria (Adedinsewo et al. 2024). The AI‐ECG algorithm result was shared with treating clinicians either through an active alert in the electronic health record or asynchronously. The follow‐up period for the primary outcome varied from 30 days to 18 months. Key characteristics of the included studies are summarized in Table 1, and the full data extraction sheet is in Data S1.

### 3.3 Quantitative Analysis

#### 3.3.1 Primary Outcome: New Diagnosis of Low Ejection Fraction (EF≤50%)

After adjusting the Yao 2021 trial for clustering (effective sample size reduced from 22,641 to 12,216), the pooled analysis of three trials showed a significant increase in the new diagnosis of low EF with AI‐ECG screening (RR = 1.36; 95% CI 1.12–1.64; *p* = 0.002), with no heterogeneity (I^2^ = 0%; *p* = 0.77) (Figure 2).

**FIGURE 2:**
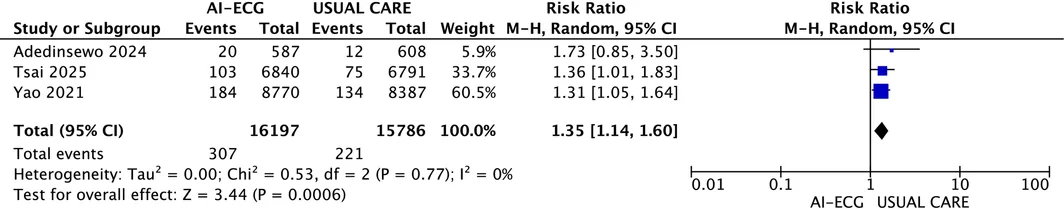
Forest plot of the primary outcome: New diagnosis of low ejection fraction (EF ≤ 50%).

The absolute risk difference corresponded to a number needed to screen (NNS) of approximately 204 patients to detect one additional case of low EF.

#### 3.3.2 Secondary Outcomes

##### 3.3.2.1 Proportion of Patients Referred for Echocardiography

After adjusting the Yao 2021 trial for clustering, the pooled analysis of two trials [11,14] showed no significant difference in echocardiography referrals between AI‐ECG screening and usual care (RR = 1.02; 95% CI 0.96–1.09; *p* = 0.55). There was low heterogeneity (*I* ^2^ = 36%; *p* = 0.21) (Figure 3). The Adedinsewo 2024 trial was excluded from this analysis because all participants in the intervention arm received a protocol‐mandated echocardiogram at baseline, which does not reflect a referral decision in routine care.

**FIGURE 3:**
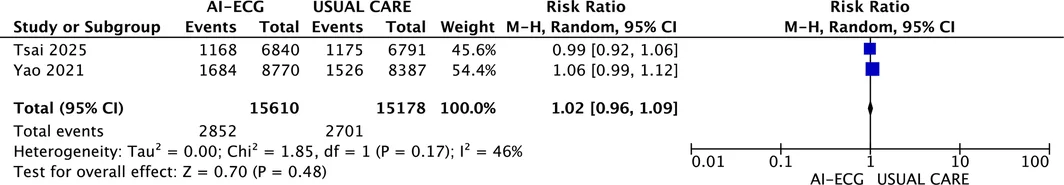
Forest plot of the secondary outcome: Proportion of patients referred for echocardiography.

##### 3.3.2.2 Positive Predictive Value (PPV) of the AI Tool

Among patients in the intervention arms who underwent echocardiography following a positive AI‐ECG screen, the PPV for low EF (≤ 50%) was 34.2% in Tsai 2025 and approximately 19.5% in Yao 2021. The necessary data to calculate PPV were not fully reported in Adedinsewo 2025.

##### 3.3.2.3 Proportion of Patients Referred to Cardiology

Only one trial [14] reported this outcome, specifically within the AI‐defined high‐risk subgroup. In that subgroup, AI‐ECG screening significantly increased the number of cardiology consultations (29.3% vs. 23.5% in usual care; *p* = 0.027). This outcome was not reported in the other two trials.

### 3.4 Certainty of the Evidence

The GRADE framework was used to evaluate the certainty of the evidence. It considers risk of bias, consistency, directness, precision, and publication bias (Table 2). For the primary outcome, new diagnosis of low ejection fraction, the evidence received a high certainty rating. This rating indicates consistent, precise results from randomized trials with a low risk of bias across key areas. These trials were conducted in populations and settings that directly apply to our situation.

**TABLE 2:** GRADE assessment of certainty of evidence.

Outcome	No. of participants (studies)	Risk of bias	Inconsistency	Indirectness	Imprecision	Publication bias	Certainty of evidence	RR (95% CI)
New diagnosis of low EF (≤ 50%)	31,983 (3 RCTs)	Serious^a^	Not serious^b^	Serious^c^	Not serious	Undetected	Low*	1.35 (1.14–1.60)
Echocardiography referral rate	30,788 (2 RCTs)	Serious^d^	Not serious	Not serious	Serious^e^	Undetected	Low*	1.02 (0.96–1.09)

*Note:* Bold text indicates the certainty of evidence as assessed by GRADE.

^a^ Downgraded one level: all three trials were rated high risk in Domain 2 (deviations from intended interventions) on RoB 2, because blinding of clinicians was not possible. Per RoB 2, a high‐risk rating in any domain yields an overall high risk of bias. The primary analysis therefore carries a serious risk of bias.

^b^ No serious inconsistency: *I* ^2^ = 0%, overlapping confidence intervals, consistent direction of effect.

^c^ Downgraded one level: the pooled primary outcome combines different EF thresholds (≤ 50% in Yao and Tsai; < 50% in Adedinsewo) and different follow‐up windows (30 days, 90 days, and through May 2024), and Adedinsewo used a combined digital stethoscope +12‐lead ECG intervention rather than AI‐ECG alone.

^d^ Downgraded one level: Yao and Tsai were both rated high risk in Domain 2 on RoB 2 for the same reason as above.

^e^ Downgraded one level: the confidence interval (0.96–1.09) crosses 1.0 and includes both a small reduction and a small increase in echocardiography referrals.

For the secondary outcome of echocardiography referral rates, the certainty was rated as moderate. This downgrade occurred because of the risk of performance bias from clinicians who were not blinded. The estimate of the effect was also imprecise, with the confidence interval ranging from a slight reduction to a modest increase in referrals. Overall, the evidence strongly supports that AI‐ECG screening improves the detection of low ejection fraction in routine care. It does this without significantly increasing echocardiographic use.

### 3.5 Risk of Bias Assessment

All three trials showed low risk of bias across randomization, missing outcome data, and selective reporting. Because the intervention was practical, blinding the clinicians was not possible. This led to a high risk of performance bias in all studies. The outcome, low ejection fraction confirmed by echocardiography, was measured objectively, resulting in a low risk of detection bias. Overall, two trials [11,14] raised concerns, mainly due to insufficient blinding. One trial [20] was assessed as having a high overall risk of bias due to performance bias and protocol‐required echocardiography in the intervention group, which did not represent a real‐world referral decision. A summary of these assessments is shown in Figure 4.

**FIGURE 4:**
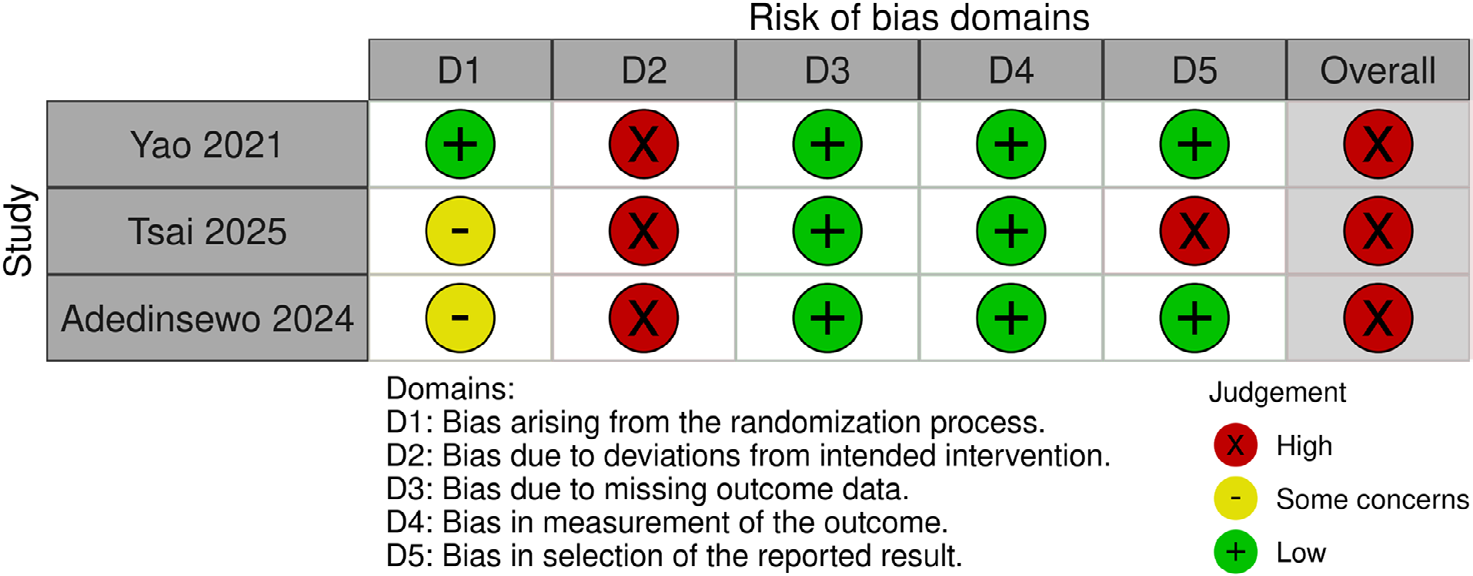
Risk of Bias Assessment of Included Randomized Controlled Trials Using the Cochrane RoB 2 Tool.

## 4 Discussion

This systematic review and meta‐analysis synthesizes evidence from three randomized controlled trials involving over 37,000 participants. The principal finding is clear and robust: integrating an AI‐ECG screening tool into routine clinical workflows significantly increases the detection of previously undiagnosed low ejection fraction by approximately 36% compared with standard care. This effect was remarkably consistent across diverse healthcare settings and populations, from primary care in the United States to hospital systems in Taiwan, with no statistical heterogeneity observed.

### 4.1 Interpretation of Key Findings and Clinical Implications

The positive effect on diagnosis occurred without a statistically significant increase in the overall rate of echocardiographic referrals in the two pragmatic trials in which referral was a clinical decision (Yao et al. 2021; Tsai et al. 2025). This suggests a critical shift in how echocardiography is used, not just an increase in volume. The AI tool seems to support a more targeted, precise approach. Clinicians, armed with a quantified probability of LVSD, may order echocardiograms with greater intent for higher‐risk patients identified by AI. This could reduce the number of “low‐yield” echocardiograms ordered based on intuition or vague symptoms in the control groups. Secondary findings from Tsai et al. support this view. They showed a significant increase in cardiology consultations, specifically among AI‐identified high‐risk patients. This indicates that specialist resources are directed toward those most likely to benefit (Tsai et al. 2025). The positive predictive values (PPV) reported in the trials (ranging from about 20% to 34%) (Yao et al. 2021; Tsai et al. 2025) are clinically meaningful, especially in a screening context. They show that one in three to five patients flagged by the AI and sent for echocardiography will have confirmed LVSD. This yield compares favorably with many other accepted screening tests in cardiology, such as systematic screening for atrial fibrillation (Svennberg et al. 2021). This efficiency is essential for ensuring the sustainability and cost‐effectiveness of such a screening program (Figures 5 and 6).

**FIGURE 5:**
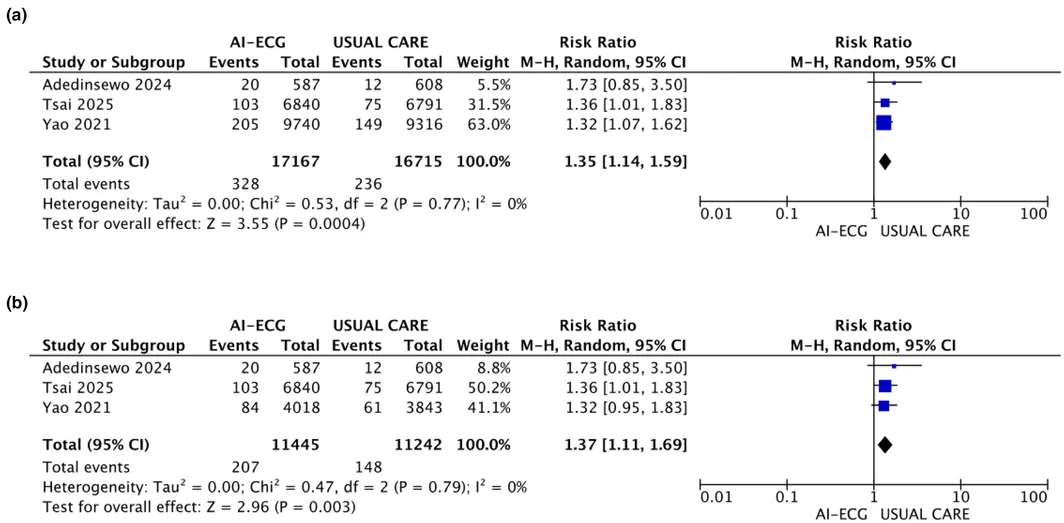
(A) Sensitivity analysis of the primary outcome (new diagnosis of low EF) using ICC = 0.001 for Yao 2021 (design effect 1.188). Pooled RR 1.35 (95% CI 1.14–1.59; *I* ^2^ = 0%). (B) Sensitivity analysis of the primary outcome (new diagnosis of low EF) using ICC = 0.01 for Yao 2021 (design effect 2.88). Pooled RR 1.37 (95% CI 1.11–1.69; *I* ^2^ = 0%).

**FIGURE 6:**
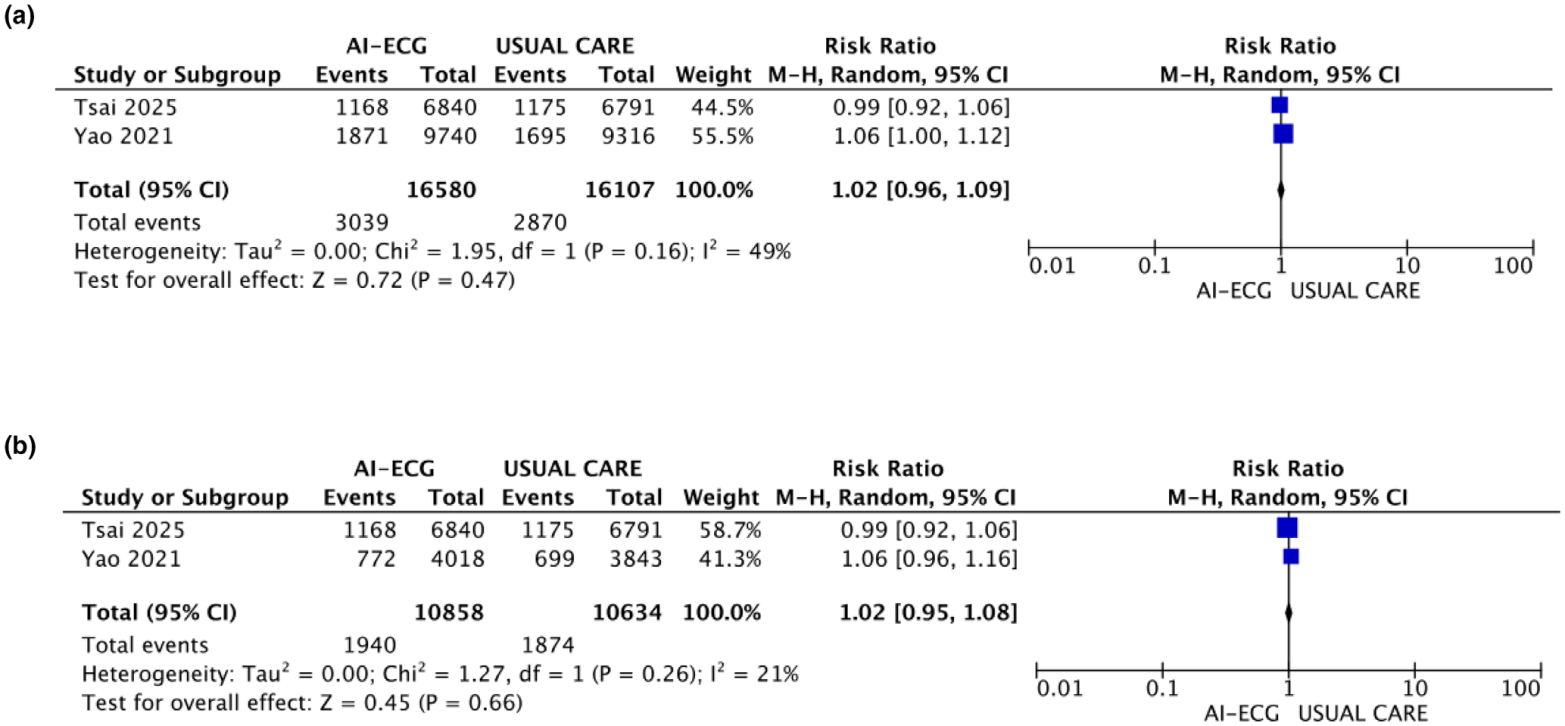
(A) Sensitivity analysis of the secondary outcome (echocardiography completion) using ICC = 0.001 for Yao 2021 (design effect 1.188). Pooled RR 1.02 (95% CI 0.96–1.09; *I* ^2^ = 49%). (B) Sensitivity analysis of the secondary outcome (echocardiography completion) using ICC = 0.01 for Yao 2021 (design effect 2.88). Pooled RR 1.02 (95% CI 0.95–1.08; *I* ^2^ = 21%).

From a broader population perspective, the number needed to screen of 204 provides additional context for implementation. This means that 204 patients need to be screened with AI‐ECG to detect one additional case of low EF that would have been missed with usual care. While this may appear high, it compares favorably with other established screening programs in cardiovascular medicine. For example, systematic screening for atrial fibrillation in older adults has reported NNS values ranging from 167 to 250 to detect one new case (Svennberg et al. 2021). Furthermore, given that the AI analysis adds minimal cost to ECGs already being performed for routine clinical indications, and considering the serious consequences of undiagnosed LVSD coupled with the availability of effective therapies, even this modest absolute yield can translate into meaningful population‐level benefits.

An important consideration when interpreting our findings is the substantial clinical heterogeneity among the three included trials. The populations differed fundamentally: Yao et al. studied older outpatients in a community healthcare system, Tsai et al. enrolled hospitalized patients in Taiwan, and Adedinsewo et al. focused exclusively on pregnant and postpartum women in Nigeria. These populations have distinct underlying pathophysiologies for left ventricular dysfunction. In the obstetric population, peripartum cardiomyopathy represents a unique disease entity with different risk factors, temporal course, and recovery patterns compared to the age‐related or ischemia‐related cardiomyopathies more common in the older outpatient and inpatient populations. Despite these differences, the direction and magnitude of effect were remarkably consistent across trials (*I* ^2^ = 0%), suggesting that AI‐ECG screening may be effective across diverse clinical contexts. However, the optimal implementation strategy, yield, and cost‐effectiveness may vary by setting. For example, the very low prevalence of LVSD in younger obstetric populations (control arm detection rate 2.0%) means that even with a similar relative increase, the absolute number needed to screen will differ substantially from older populations. We have made these differences transparent in Table 1, and we encourage readers to consider how their local population characteristics might influence the expected benefit of AI‐ECG screening.

### 4.2 Integration With Existing Evidence and Biological Plausibility

Our findings connect previous studies on diagnostic accuracy and real clinical impact. They show that the subtle changes in heart function caused by LVSD, which AI models can detect, provide a reliable signal to assist in real‐world clinical decisions (Attia et al. 2019; Hughes et al. 2021). The success of this intervention follows ideas from behavioral economics and implementation science. The AI results act as a “nudge,” lowering diagnostic uncertainty and encouraging action for a condition where doctors often hesitate (Saposnik et al. 2016).

The results also add to the growing research on AI in cardiovascular medicine, broadening the focus beyond LVSD detection to include the identification of atrial fibrillation, hypertrophic cardiomyopathy, and valvular heart disease from ECGs (Raghunath et al. 2021; Ko et al. 2020). The potential of AI‐ECG extends beyond detection to serve as a rule‐out tool, a concept well‐illustrated by the recent ROMIAE multicentre study, which demonstrated that an AI‐ECG algorithm could safely exclude acute myocardial infarction in emergency department patients, potentially reducing unnecessary admissions and testing (Lee et al. 2025). This paradigm shift, using AI to rule out disease rather than just flag it, may be equally applicable to LVSD screening. The consistently high negative predictive values (> 95%) observed across the included trials suggest that a negative AI‐ECG result could reasonably exclude clinically significant ventricular dysfunction, offering a strategy to reduce low‐yield echocardiography while maintaining patient safety. This meta‐analysis provides some of the strongest evidence to date that this technology can leave the lab and make a real difference in diagnosing a life‐changing condition.

### 4.3 Considerations for Implementation and Equity

Successful implementation requires careful attention to workflow. The included trials used either passive integration into the electronic health record or asynchronous notification (Yao et al. 2021; Tsai et al. 2025; Adedinsewo et al. 2024). The best way to make sure alerts are noticed and acted on without causing alarm fatigue is still an area for operational research (Sutton et al. 2020). Additionally, the performance of AI models can differ across various patient demographics and ECG acquisition devices (Adedinsewo et al. 2020). Recent multinational head‐to‐head comparisons have highlighted the importance of assessing AI‐ECG performance across independent datasets and algorithmic architectures, reinforcing the need for rigorous local validation before widespread implementation (Croon et al. 2026). An additional consideration is the lack of interpretability of AI‐ECG models, which derive predictions from raw waveform patterns without providing human‐interpretable features. This ‘black box’ nature may limit clinician trust and adoption, highlighting the need for explainable AI approaches that can provide insights into which ECG characteristics drive the algorithm's decision. The model used in the Nigerian trial, for example, performed differently in younger, pregnant women. This emphasizes the need for external validation in specific populations and ongoing monitoring for performance changes (Adedinsewo et al. 2024; Noseworthy et al. 2022). To ensure health equity, we must thoroughly test these tools across different racial, ethnic, and gender groups. This helps prevent algorithmic bias from worsening existing care disparities (Obermeyer et al. 2019).

### 4.4 Strengths, Limitations, and Future Directions

A key strength of this review is its exclusive focus on RCTs, the highest level of evidence for evaluating an intervention's effect. The use of a random‐effects model and the pre‐specified sensitivity analysis strengthen the robustness of our conclusions.

Limitations must be acknowledged. First, the follow‐up periods were relatively short, ranging from 30 days to 18 months. While we observed an increase in diagnoses, our main goal is to improve long‐term outcomes, such as heart failure, hospitalizations, and mortality. Future trials with longer follow‐up are needed to see if earlier diagnosis supported by AI leads to earlier treatment and better prognosis (Seferović et al. 2019). Second, one included trial involved a specific group of pregnant and postpartum women, along with a protocol‐driven echocardiography design. This limitation reduced its comparability for the referral outcome (Adedinsewo et al. 2024). Third, the included trials represent clinically heterogeneous populations, outpatients, inpatients, and obstetric patients, with distinct underlying pathophysiologies for left ventricular dysfunction. While statistical heterogeneity was low (*I* ^2^ = 0% for the primary outcome), this clinical diversity means that pooled estimates should be applied cautiously to specific settings, and the optimal implementation strategy may vary by population. Fourth, data on follow‐up management, such as starting guideline‐directed medical therapy, were reported inconsistently. This inconsistency made it impossible to conduct a meta‐analysis on this important step in the care pathway. Fifth, while our inclusion criteria specified patients without known heart failure, we cannot confirm that all participants were truly asymptomatic. The included trials did not systematically assess or exclude patients based on symptom status, and some participants may have had symptoms consistent with undiagnosed heart failure. Therefore, our findings apply to patients undergoing routine ECGs in clinical care who do not have a prior diagnosis of LVSD, rather than to a strictly asymptomatic screening population. This distinction is important for implementation, as the yield of screening may differ in truly asymptomatic versus symptomatic but undiagnosed populations. Fifth, two of the three included trials [11,20] used AI algorithms derived from the same platform lineage (Mayo Clinic), while only one trial [14] evaluated an independently developed model. This limits the generalizability of our findings to all AI‐ECG platforms, and reproducibility across different developers, datasets, and device environments remains to be established. Additionally, the included trials had varying follow‐up periods (ranging from 30 days to 18 months), which could theoretically influence detection rates. While a formal time‐to‐event analysis was not possible due to the binary nature of the outcome data and lack of patient‐level data, the consistency of effects across studies (*I* ^2^ = 0%) and the absence of a clear relationship between follow‐up duration and effect size suggest this did not materially impact our findings. Finally, the small number of included studies (*n* = 3) limited our ability to assess publication bias or perform meaningful subgroup analyses, and the confidence intervals around our pooled estimates, while statistically significant, are relatively wide. Moreover, while we observed increased detection of LVSD, this does not necessarily equate to improved clinical outcomes, and the possibilities of overdiagnosis and lead‐time bias cannot be excluded without longer‐term outcome data.

Future research should focus on longer‐term outcome trials. We also need cost‐effectiveness analyses to guide policy and reimbursement decisions (Khera et al. 2020). Looking into how to combine AI‐ECG with other point‐of‐care tools, such as handheld ultrasound, offers a promising way to create effective and accessible diagnostic paths (Narula et al. 2016). Finally, as AI becomes a larger part of care, we need to develop ethics frameworks for its use, provide clinicians with education, and maintain the human aspects of the patient‐clinician relationship (Gerke et al. 2020; Meskó and Görög 2020).

## 5 Conclusion

In conclusion, adding AI‐ECG screening to routine care significantly improves the detection of low ejection fraction without substantially increasing echocardiography referrals. These findings support AI‐ECG as an effective screening tool, though further validation across diverse populations and AI platforms, as well as longer‐term outcome data, are needed to confirm its impact on patient prognosis.

## Author Contributions

A.A.O.A.: conceptualization, methodology, project administration, writing – original draft, writing – review and editing, formal analysis. O.A.O.A.: data curation, formal analysis, validation. R.A.O.A. contributed to data curation, investigation, and visualization. A.S.D. participated in investigation and validation. C.G. assisted in methodology development and validation. M.M.A.M. was responsible for data curation and resource provision. Y.A. contributed to visualization and investigation. M.H.A. handled data curation, resource management, and validation. A.A.E. provided supervision and critical review of the manuscript. All authors read and approved the final version of the manuscript.

## Funding

The authors have nothing to report.

## Ethics Statement

The authors have nothing to report.

## Consent

The authors have nothing to report.

## Conflicts of Interest

The authors declare no conflicts of interest.

## Supporting information


**Data S1:** Data Extraction Sheet. Standardized form used to record study characteristics, participant demographics, intervention and comparator details, and outcome data from all included randomized controlled trials.

## Data Availability

The datasets used and/or analyzed during the current study are available from the corresponding author on reasonable request. The complete data extraction sheet is provided as Data S1.
